# Metronidazole is an effective method of analgesia following haemorrhoidectomy: a systematic review and meta-analysis

**DOI:** 10.1007/s00384-025-05066-7

**Published:** 2026-01-26

**Authors:** Aaron O’Mahony, Carolyn Cullinane, Benjamin M. MacCurtain, Colin Peirce, Eoghan Condon, J. Calvin Coffey, Christina A. Fleming

**Affiliations:** 1 Department of Colorectal Surgery, University Hospital Limerick, Limerick, Ireland; 2 Department of Surgery, University Hospital Waterford, Waterford, Ireland; 3 Department of Surgery, Royal College of Surgeons Ireland, Dublin, Ireland; 4 School of Medicine, University of Limerick, Limerick, Ireland

**Keywords:** Metronidazole, Haemorrhoids, RCT

## Abstract

**Background:**

Haemorrhoids are one of the most frequently encountered benign anorectal conditions that negatively impact patients’ quality of life. Excisional haemorrhoidectomy (closed or open) is a surgical procedure reserved for the treatment of third- and fourth-degree haemorrhoids, with considerable post-procedure pain reported. The aim of this study was to clarify the association between post operative metronidazole use (both oral and topical) and post-haemorrhoidectomy pain scores through systematic review and meta-analysis of randomised controlled trials (RCTs).

**Methods:**

This study was guided by the Preferred Reporting Items for Systematic Reviews and Meta-Analysis (PRISMA) guidelines. Prospective registration was performed on PROSPERO (CRD42024580928). A systematic review was performed for RCTs reporting post-haemorrhoidectomy pain scores between patients who received metronidazole and patients who received placebo. Meta-analysis was performed using RevMan version 5.4.

**Results:**

Seventeen RCTs including 1297 participants were eligible for inclusion. Metronidazole administration was associated with significantly lower post-operative visual analogue scores (VAS) on day 1 (−1.18, *p* < 0.00001), day 2 (−1.15, *p* = 0.003), day 3 (−0.86, *p* < 0.00001), and day 7 post-operatively with a mean pain score difference of −1.72 (95% CI −2.27 to −1.18) (*p* < 0.00001). A significant difference in pain scores was seen on day 3 favouring topical metronidazole in comparison to the oral route (1.38, 95% CI [0.44, 2.32], *p* = 0.004).

**Conclusion:**

This review synthesises the best available evidence to support the use of metronidazole to reduce pain after excisional haemorrhoidectomy. While both oral and topical forms appear to be beneficial, topical administration appears to have a more effective analgesic effect from post-operative day 3.

**Supplementary Information:**

The online version contains supplementary material available at 10.1007/s00384-025-05066-7.

## Introduction

Haemorrhoids are one of the most frequently encountered benign anorectal conditions. There is a significant variation in the reported prevalence of haemorrhoids ranging from 13% up to 36% depending on the population studied [1, 2]. Symptoms vary from pruritus and minor bleeding to thrombosis and severe pain, often leading to a marked reduction in quality of life and a significant healthcare burden. Excisional haemorrhoidectomy remains the standard treatment for advanced disease but is associated with severe post-operative pain, reported in up to 65% of cases [3–5].

Effective peri-operative analgesia is therefore critical. Conventional strategies include oral analgesics, local anaesthetic infiltration, laxatives, and topical nitroglycerine, but their efficacy is variable [6]. Metronidazole (Flagyl®), an antibiotic and antiprotozoal, has emerged as a promising analgesic agent post-haemorrhoidectomy [7]. The antioxidant properties possessed by metronidazole are believed to alleviate oxidative stress and, consequently, reduce pain. Another plausible theory is that metronidazole exhibits potent antibacterial activity against anaerobic gut organisms which colonise the wound. These organisms could potentially hinder wound healing [8].

In the last two decades, several studies have demonstrated that oral metronidazole is an effective pain management strategy following haemorrhoidectomy [7, 9]. Previous systematic reviews have provided conflicting conclusions, limited by the inclusion of only open haemorrhoidectomy, exclusion of topical preparations, and omission of more recent RCTs reflecting contemporary practice. Furthermore, no prior meta-analysis has directly compared oral and topical administration.

This systematic review and meta-analysis was conducted to evaluate the effect of oral and topical metronidazole on post-haemorrhoidectomy pain scores, synthesising the most up-to-date randomised evidence and directly comparing routes of administration.

## Methods

### Search strategy

The search was conducted in line with the most recent Preferred Reporting Items for Systematic reviews and Meta-Analyses (PRISMA) recommendations [10]. Our study protocol was prospectively registered with PROSPERO under the following registration number: CRD42024580928. A search of PubMed, EMBASE, and Cochrane Central Register of Controlled Trials was conducted in September 2025. The search strategy can be found below:

[Embase: (“metronidazole”/exp OR “metronidazole”) AND (“hemorrhoid”/exp OR “hemorrhoid” OR “hemorrhoidectomy”/exp OR “hemorrhoidectomy” OR “anorectal surgery”/exp OR “anorectal surgery” OR “perianal surgery”/exp OR “perianal surgery”) AND (“pain”/exp OR “pain” OR “analgesia”/exp OR “analgesia”)]; [Pubmed: ((((metronidazole[MeSH Terms]) OR (flagyl[MeSH Terms])) AND (anorectal surgery[MeSH Terms]) OR (haemorrhoidectomy[MeSH Terms])) OR (haemorrhoid[MeSH Terms] OR hemorrhoidectomy[MeSH Terms])) AND (pain[MeSH Terms])]; [Cochrane: metronidazole AND (hemorrhoidectomy OR hemorrhoids OR anorectal surgery OR perianal surgery) AND (pain OR analgesia)].

### Inclusion criteria

Randomised controlled trials that met the following criteria were considered for inclusion: patients aged 18 years or above, studies written in the English language or suitable translation available, studies including patients who underwent haemorrhoidectomy and received treatment with oral and/or topical metronidazole following same, studies including open and/or closed haemorrhoidectomy, and studies reporting pain scores following haemorrhoidectomy.

### Exclusion criteria

The following studies were excluded from analysis: non-randomised studies, prospective cohort studies, retrospective cohort studies, case reports, case series, conference abstracts, and systematic reviews. Studies with patients who underwent haemorrhoidal banding or injections were excluded, as were studies where no suitable English translation or full text was available. Studies where alternative analgesia to metronidazole was used, e.g. sucralfate and nerve block, or where pain scores from either oral or topical metronidazole alone could not be compared with each other or placebo were also not included.

#### Study selection

**Fig. 1:**
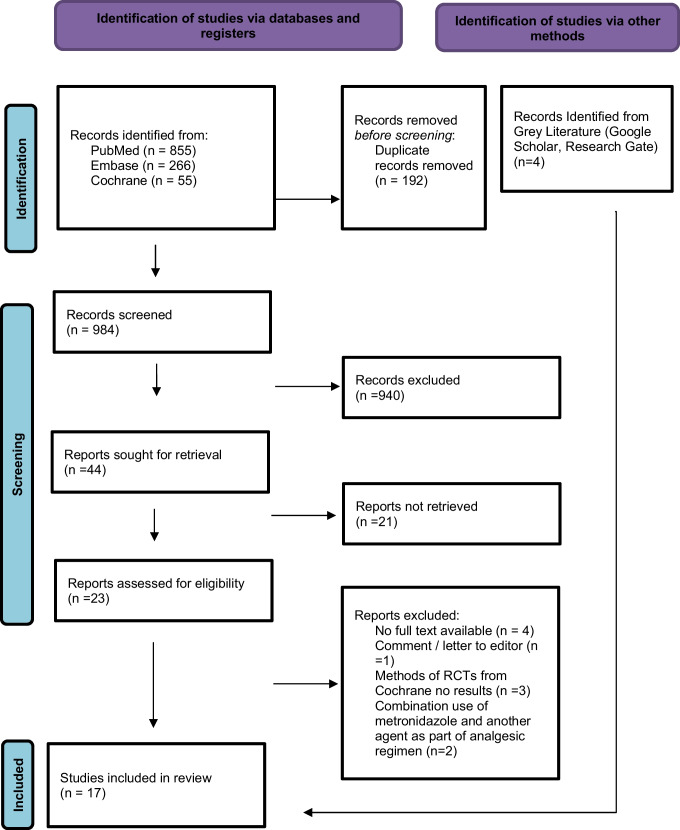
PRISMA flow diagram of included studies

Studies were independently reviewed using the online systematic review tool Rayyan [11] by two separate authors (AOM, BMC). Discordance regarding articles to be included for review was reviewed by a third author (CC) until an agreement was reached. A grey literature review was also conducted by one of the authors (AOM) in order to identify any studies which may have fallen outside of the scope of the search study parameters, or that may not be found on the traditional commercial and academic databases. After identifying studies which met the inclusion criteria, these papers were discussed amongst the other authors (BMC, CC), deemed of satisfactory quality and therefore were included in this review. A summary PRISMA flow diagram of the study selection can be found below in Figure 1.

#### Outcomes

The primary outcome was to determine whether peri-operative metronidazole administration reduced haemorrhoidectomy post-operative day one (POD 1) pain scores. The most widely used pain-intensity assessment measure that is used in academia is the visual analogue score (VAS). It is usually presented as a 10 cm horizontal line on which the patient’s pain intensity is represented by a point between the extremes of “no pain at all” and “worst pain imaginable.” Its simplicity, reliability, validity, and ratio scale properties make the VAS an attractive tool for describing pain severity [12]. Secondary outcomes included post-operative day 2 (POD2), day 3 (POD3), and day 7 (POD7) pain scores between patients who received metronidazole and those who did not. Subgroup analysis was performed to assess whether oral or topical administration of metronidazole was associated with reduced post-operative pain scores.

#### Data extraction

Data extraction was conducted by three authors (AOM, BMC, CC). A template was formed with the relevant headings, and metrics were extracted from the included studies and transcribed onto the templated table using Google Sheets (Alphabet, Mountain View, CA, USA). Google Sheets was used to allow all authors to update the table in real time and edit accordingly (Table 1).

**Table 1:** Characteristics of included studies (*VAS visual analogue score, *N number)

Author	Year	Country	Sample size	Age (mean)	Oral or topical	Control	N study group	N control	Type of surgery	Pain score
Wilkie [17]	2021	Australia	40	45	Oral	Placebo	21	19	Both	Likert Scale 0 to 10
Carapeti [7]	1998	UK	40	49	Oral	Placebo	20	20	-	Likert Scale 0 to 10
Balfour [18]	2002	UK	38	56	Oral	Placebo	18	20	Closed	Likert Scale 0 to 10
Nicholson [19]	2004	USA	20	48	Topical	Placebo	10	10	Closed	VAS 0 to 10
Ala [20]	2008	Iran	47	37	Topical	Placebo	25	22	Open	VAS 0 to 10
Ng [21]	2006	Hong Kong	52	49.35	Oral	Placebo	26	26	Open	VAS 0 to 10
Rabelo [22]	2021	Brazil	34	42	Oral	Placebo	17	17	Open	VAS 0 to 10
Solorio-López [9]	2015	Mexico	44	46	Oral	Placebo	22	22	Closed	VAS 0 to 10
Xia [23]	2022	New Zealand	120	45	Both	Group A Topical Group B Oral	60 Topical	60 Oral	Both	VAS 0 to 10
Neogi [24]	2018	India	60	-	Both	Group A Topical, Group B Oral, Group C Control	20 Topical 20 Oral	20 Control	Open	VAS 0 to 10
Abbas [25]	2020	Pakistan	166	43.5	Both	Group A Topical Group B Oral	83	83	Open	VAS 0 to 10
Munawar [26]	2023	Pakistan	140	41.9	Oral	Placebo	70	70	Open	VAS 0 to 10
Razzaq [27]	2020	Pakistan	120	38.74	Both	Group A Topical, Group B Oral	60	60	Open	VAS 0 to 10
Ghumro [28]	2023	Pakistan	60	37.3	Oral	Placebo	30	30	Open	VAS 0 to 10
Al-Mulhim [29]	2006	Saudi Arabia	166	47	Oral	Placebo	84	82	Open	VAS 0 to 10
Abdullah [30]	2024	India	90	51.7	Both	Group A Topical, Group B Oral	45	45	Open	VAS 0 to 10
Firdous [31]	2025	Pakistan	60	45.25	Both	Group A Topical, Group B Oral	30	30	Open	VAS 0 to 10

#### Risk of bias

As this study included only RCTs, the Cochrane Risk of Bias Tool (RoB-2), for assessing risk of bias in randomised controlled trials, was utilised [13]. The risk of bias was conducted by two authors (AOM and BMC) independently. The RoB-2 tool grades papers for bias risk assessment as low risk, some concerns, or high risk. The RoB-2 risk of assessment is outlined in Table 2.

**Table 2:**
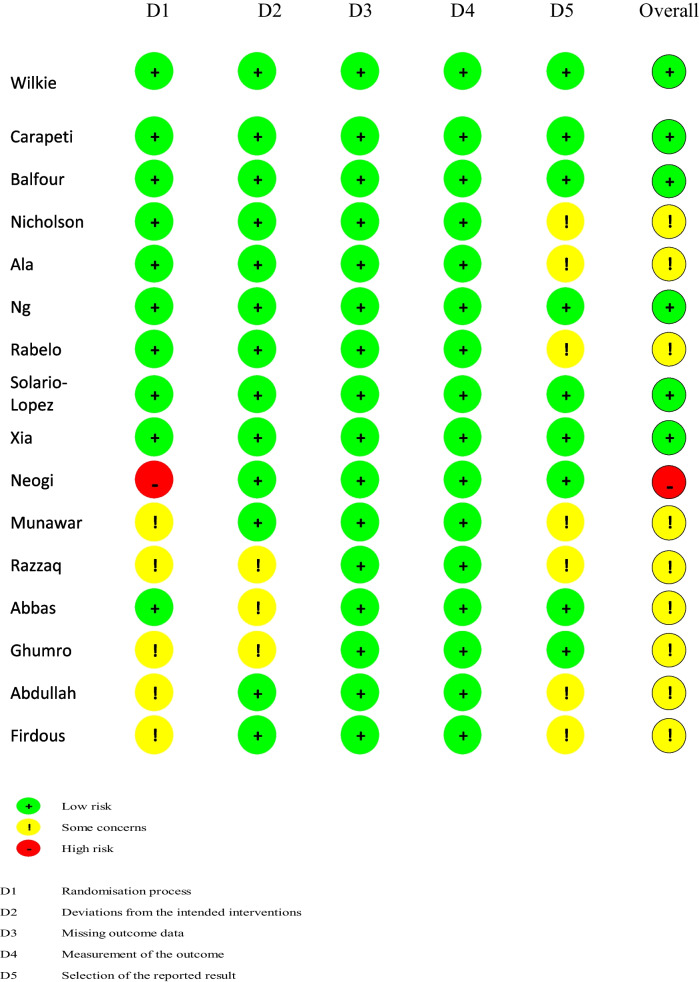
Summary of Rob-2 Results

#### Statistical analysis

Statistical analysis was performed using Review Manager 5.4 (RevMan). Continuous outcome data were reported as mean values and standard deviation (SD). The mean values and SD reported in the study publication were used when available; otherwise, they were extrapolated from the available data. If the standard error of the mean (SEM) was reported, it was converted to standard deviation using the formula SD = SEM × (√n) as outlined in the Cochrane Handbook of Systematic Reviews [14]. If the values were reported as a median and the full range or interquartile range was given, the mean was calculated using the formula as described by Luo [15], and the standard deviation estimated as per Wan [16]. RCTs without a reported standard deviation (SD) in their results were excluded from the meta-analysis. VAS and Likert scales were considered akin due to both scales ranging from 0 to 10, with the same extremes of “no pain at all” and “worst pain imaginable.” Weighted mean differences (MDs) were calculated for the effect size on continuous variables. Heterogeneity was assessed by using *I*^2^ statistics. Given the inherent heterogeneity between nonrandomised studies, a random-effects model was used. Pooled estimates of differences were calculated using a random-effects model, accounting for potential interstudy heterogeneity. A *p*-value of < 0.05 was considered significant. A random-effects model was also used in previous systematic reviews with similar data to the studies included in this review. The mean pain scores were extracted from POD1, POD2, POD3, and POD7 as these were the most frequently reported across the RCTs. All corresponding authors of the studies were contacted to provide any missing information encountered.

## Results

Seventeen RCTs with 1297 participants were included in this systematic review and meta-analysis. The mean age of the participants across the trials was 45.2 ± 5.15 (SD). Characteristics of the included studies are outlined in Table 1. One study from Balfour and colleagues [18] did not provide any standard deviation in their manuscript and thus was excluded from our meta-analysis. Three of the RCTs used generic Likert scales [7, 17, 18] to report post-operative pain scores, whilst the rest of the RCTs utilised VAS to assess and report pain. Nine studies investigated the use of oral metronidazole with a placebo as the control group [7, 9, 17, 18, 21, 22, 26, 28, 29], and two studies examined the use of topical metronidazole versus placebo [19, 20]. Five studies compared oral against topical metronidazole [23, 25, 27, 30, 31], whilst Neogi compared oral metronidazole, topical metronidazole, and placebo [24]. The majority of the studies in this review solely focused on patients post Milligan–Morgan haemorrhoidectomy [20, 21, 25–31]. Nicholson, Balfour, and Solorio-Lopez only included patients who underwent the closed (Ferguson) technique [9, 18, 19]. Xia and Wilkie included both techniques in their study cohorts, and Carapeti et al. did not clearly outline what surgical technique or approach was adopted [7, 17, 23]. The sample sizes of the included studies were relatively small, with a range from 20 to 200 [19, 29]. Seven of the studies included 50 participants or less [7, 9, 17–20, 22].

Overall, the risk of bias across the included trials was variable, with many studies demonstrating methodological limitations. While several RCTs were assessed as having low risk of bias in multiple domains, more than half were judged to have “some concerns,” primarily due to insufficient detail regarding randomisation procedures. Additionally, selective reporting could not be excluded in several trials owing to limited availability of protocols. One study demonstrated a high risk of bias in relation to its randomisation process [24].

### Overall metronidazole vs. placebo

**Fig. 2:**
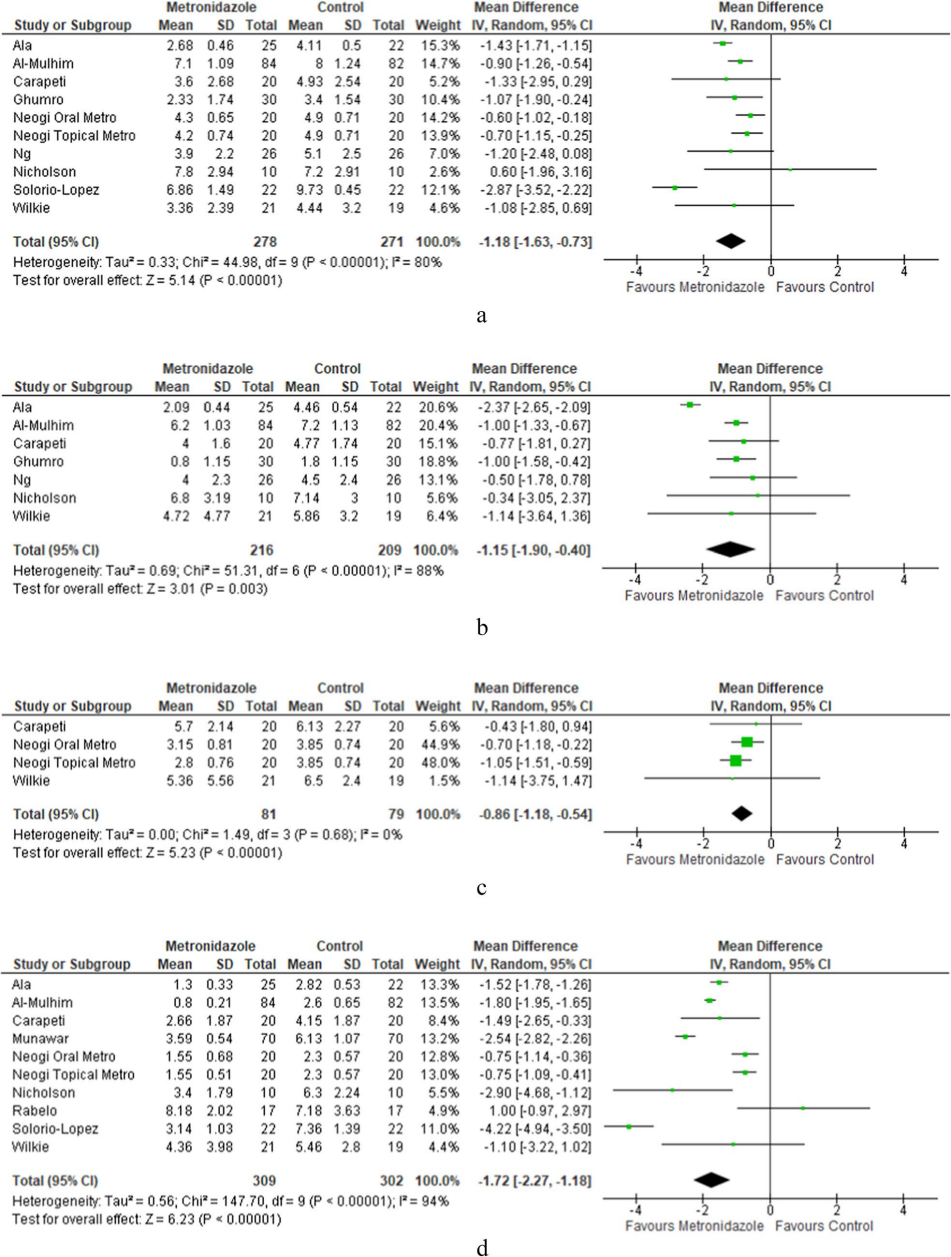
**a **Overall comparison of POD 1 VAS scores between metronidazole and control. **b** Overall comparison of POD 2 VAS scores between metronidazole and control. **c **Overall comparison of POD 3 VAS scores between metronidazole and control. **d** Overall comparison of POD 7 VAS scores between metronidazole and control

Ten study subgroups examined post-operative pain scores amongst patients who received metronidazole (either oral or topical) and those who received a control [7, 9, 17, 19–21, 24, 28, 29]. Metronidazole administration was associated with significantly lower post-operative VAS scores on day 1 (MD = −1.18, 95% CI [–1.63, –0.73], *p* < 0.00001), day 2 (MD = −1.15, 95% CI [–1.9 to –0.4], *p* = 0.003), and day 3 (MD = −0.86, 95% CI [–1.18, –0.54], *p* < 0.00001) (Fig. 2a–c). The largest effect size was seen on day 7 with a mean pain score difference of −1.72 (95% CI [–2.27, –1.18], *p* < 0.00001) (Fig. 2 d). There was a significant degree of heterogeneity between the studies (*I*^2^ = 80%, 88%, and 94% on days 1, 2, and 7 respectively).

#### Oral metronidazole vs. placebo

**Fig. 3:**
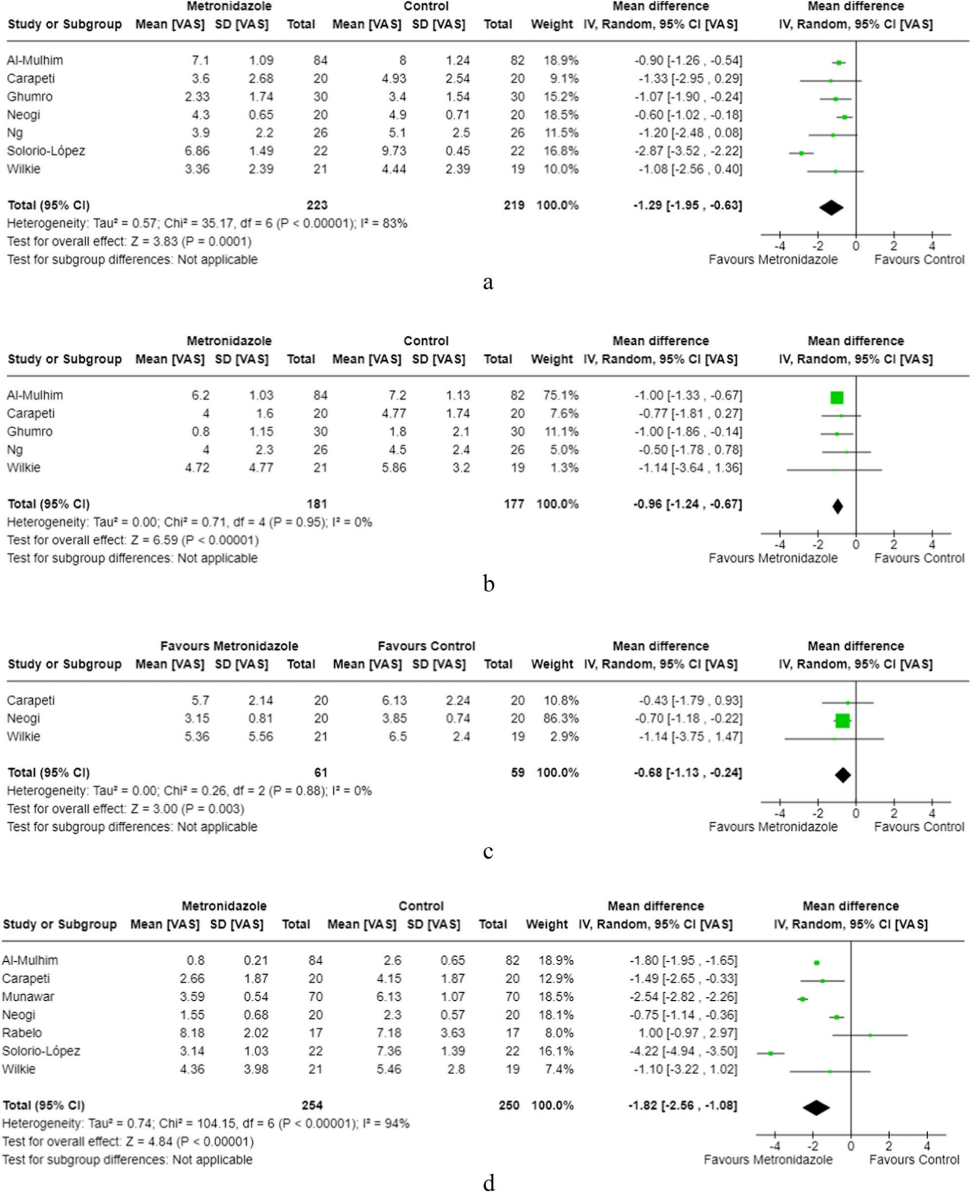
**a **Oral metronidazole day 1 post-op pain scores. **b** Oral metronidazole day 2 post-op pain scores. **c **Oral metronidazole day 3 post-op pain scores. **d** Oral metronidazole day 7 post-op pain scores

Seven of the included studies [7, 9, 17, 24, 28, 29] compared oral metronidazole to control and reported significantly lower VAS scores in the oral metronidazole cohorts on day 1 (−1.29, 95% CI [−1.95, −0.63], *p* = 0.0001), day 2 (−0.96, 95% CI [−1.24, −0.67], *p* < 0.00001), day 3 (−0.68, 95% CI [−1.13, −0.24], p = 0.03), and day 7 (−1.82, 95% CI [−2.56, −1.08], *p* < 0.0001) post-haemorrhoidectomy (Fig. 3a–d).

#### Topical metronidazole vs. placebo

**Fig. 4:**
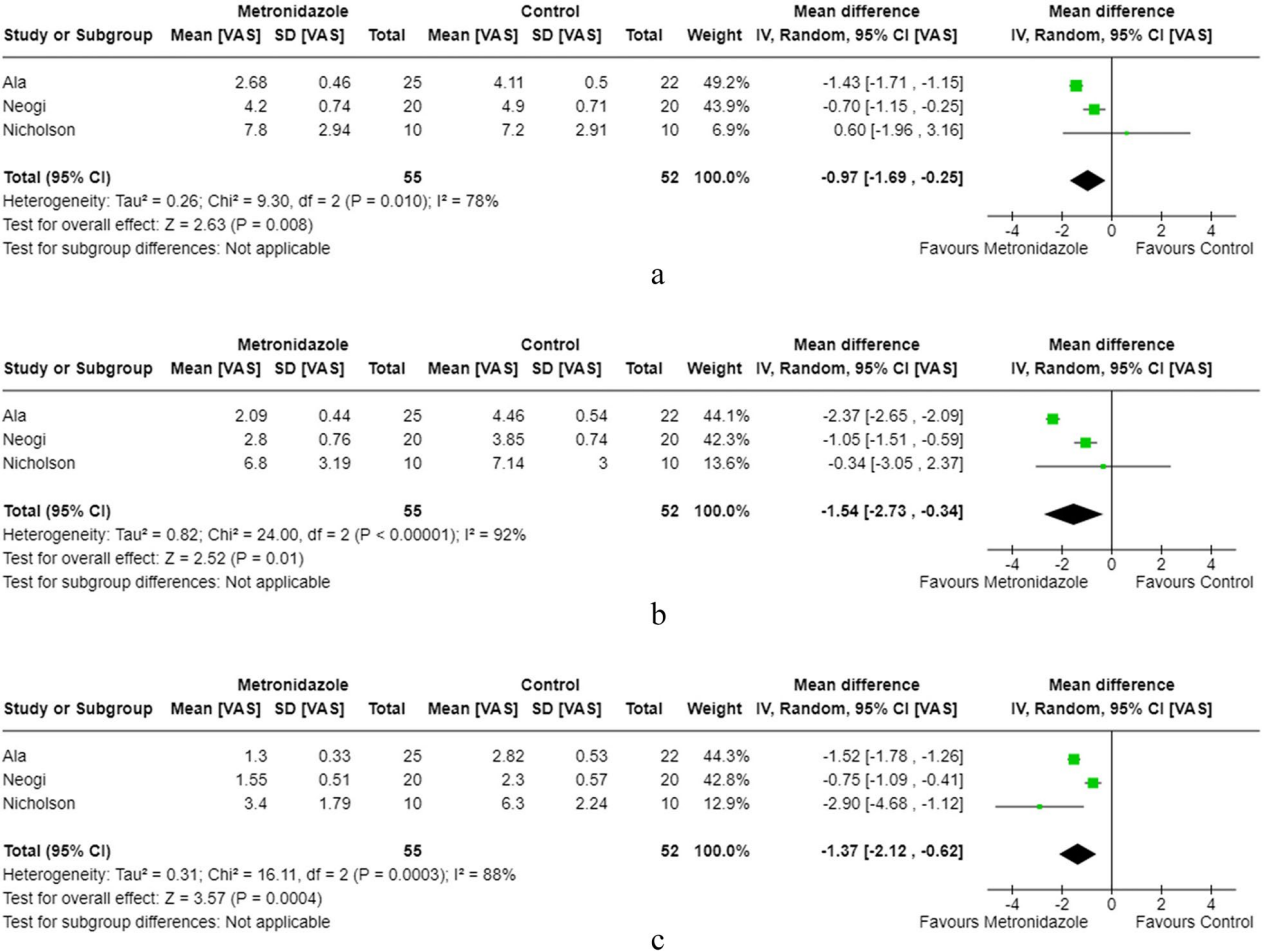
**a** Topical metronidazole day 1 post-op pain score. **b** Topical metronidazole day 2/3 post-op pain score. **c** Topical metronidazole day 7 post-op pain score

Three studies compared the effect of topical metronidazole to placebo in terms of post-haemorrhoidectomy VAS scores [19, 20, 24]. Topical metronidazole was associated with lower VAS scores on day 1 (–0.97, 95% CI [–1.69, –0.25], *p* = 0.008), day 2–3 (–1.54, 95% CI [–2.73, –0.34], *p *= 0.01), and day 7 (–1.37, 95% CI [–2.12, –0.62], *p* = 0.0004) with significant heterogeneity between all studies (Fig. 4a–c).

#### Oral vs. topical metronidazole

**Fig. 5:**
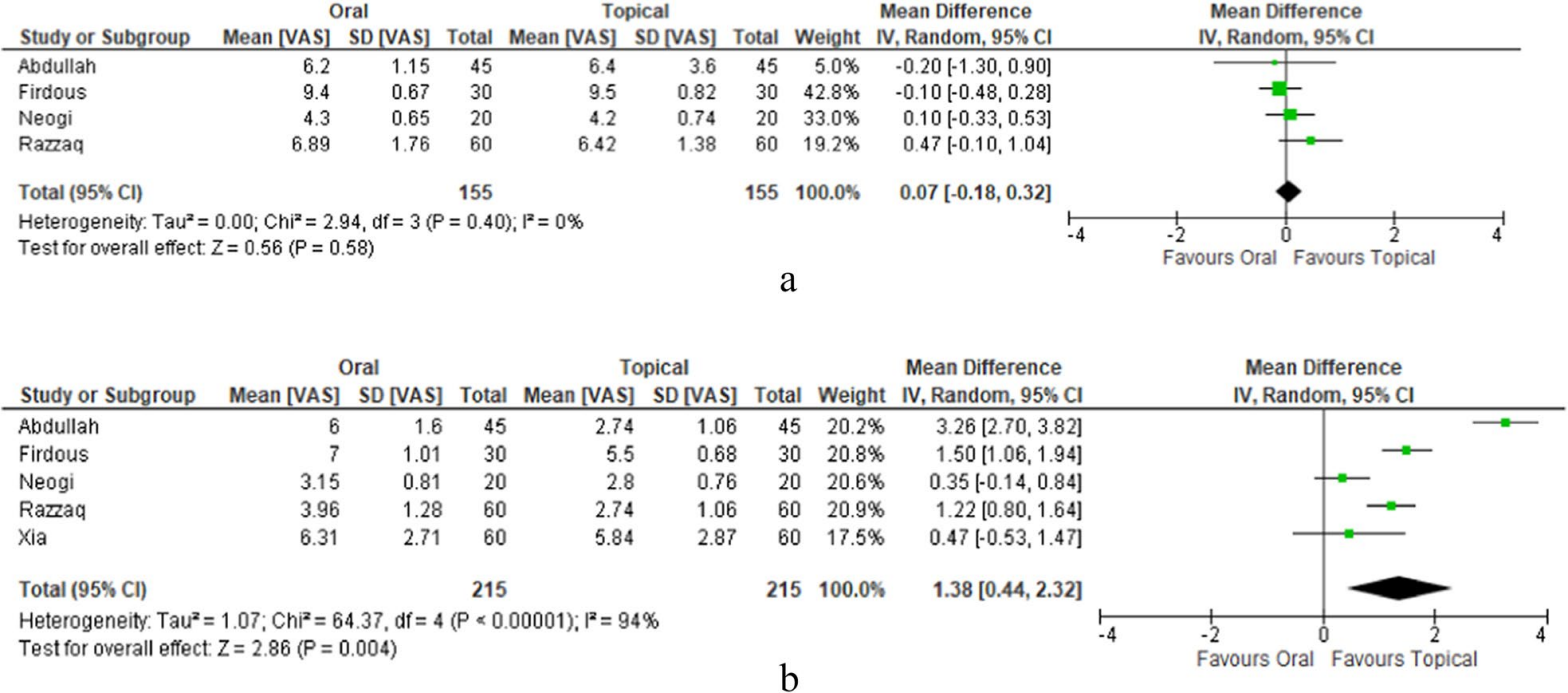
**a** Oral metronidazole vs. topical metronidazole day 1 post-op pain score. **b** Oral metronidazole vs. topical metronidazole day 3 post-op pain score

Six compared post-haemorrhoidectomy VAS scores in oral metronidazole and topical metronidazole cohorts [23–25, 27, 30, 31]. There was no significant difference in VAS scores on day 1 post-haemorrhoidectomy between the two cohorts (0.07, 95% CI [− 0.18, 0.32], *p* = 0.58) (Fig. 5a). However, there was a statistically significant reduction seen in pain scores on post-operative day 3, favouring the topical administration of metronidazole (1.38, 95% CI [0.44, 2.32], *p* = 0.004). There was substantial heterogeneity amongst the included studies (chi^2^ = 64.37, df = 4, *p* < 0.00001; *I*^2^ = 94%) (Fig. 5b).

## Discussion

This meta-analysis of 17 RCTs demonstrates that metronidazole significantly reduces pain after excisional haemorrhoidectomy compared with placebo, with benefits observed from POD1 through POD7. The largest effect was seen by day 7 (MD −1.72, (95% CI [–2.27, –1.18], *p* < 0.00001)) indicating a sustained analgesic benefit. Subgroup analysis showed that topical administration provides superior pain relief compared with oral metronidazole up to POD3 (1.38, 95% CI [0.44, 2.32], *p* = 0.004), suggesting a potential early advantage of local therapy. In the era of drug repurposing, metronidazole has emerged as an attractive pharmacological solution as an alternative analgesic.

Metronidazole’s analgesic mechanism of action is not clearly understood, but the prevailing theory centres on the presence of bacterial colonisation after haemorrhoidectomy. The change in microbiota is believed to delay wound healing, incite inflammation, and cause significant pain [32]. The mechanisms underlying metronidazole’s analgesic effect remain uncertain. Proposed explanations include suppression of anaerobic wound colonisation, attenuation of inflammatory pathways, and possible antioxidant activity. Experimental data support an immunomodulatory role, with inhibition of pro-inflammatory cytokines such as IL-1, IL-6, IL-8, and TNF-α. These properties may reduce mucosal inflammation and promote wound healing, thereby lowering nociceptive input [33]. The anti-inflammatory effects of metronidazole in periodontitis have been widely studied [34]. Given the similarities between the oral cavity and gastrointestinal mucosal flora, it would be reasonable to deduce that metronidazole exhibits a similar effect in the anal canal to decrease wound colonisation, reducing bacterial growth, perhaps leading to a reduction in inflammation and pain [9].

Oral metronidazole can cause gastrointestinal upset, metallic taste, and, rarely, neurotoxicity. Our review suggests that topical administration is more effective whilst potentially avoiding these systemic side effects.

Due to dependency concerns, non-opioid painkillers are continually explored with significant benefits. The opioid epidemic is one of the western worlds’ biggest public health crises [35]. Unfortunately, patients following haemorrhoidectomy typically require varying quantities of narcotics to alleviate pain. Several of the clinical trials included in this meta-analysis demonstrated that metronidazole administration significantly reduced opioid use amongst participants [19, 20]. Strategies that reduce opioid consumption are valuable. Metronidazole, especially in topical form, may therefore represent a safe, cost-effective adjunct to multimodal analgesia protocols.

Our findings differ from the 2017 meta-analysis by Wanis et al., which reported no benefit of oral metronidazole [36]. That review was limited by exclusion of topical preparations, reliance on only five small RCTs, and disproportionate weighting of a single large study. Since then, several additional trials have been published, providing a more robust evidence base.

The updated 2023 guidelines by the PROSPECT Working Group of the European Society of Regional Anaesthesia and Pain Therapy (ESRA) advocate a multimodal analgesic approach following haemorrhoidectomy, recommending topical metronidazole as part of a combined postoperative analgesic strategy [37]. Interestingly, the same group previously recommended the use of oral metronidazole following excisional haemorrhoidectomy [38]. This reflects a broader shift toward layered, opioid-sparing regimens in anorectal surgery. The recent TAPH trial further examined whether a multi-agent topical approach might enhance analgesia but demonstrated no additional benefit from adding diltiazem or lidocaine to topical metronidazole, suggesting that metronidazole alone may provide the principal effect [39]. Several of the clinical trials in this meta-analysis also reported that metronidazole use was associated with a meaningful reduction in postoperative opioid consumption [19, 20], underscoring its potential value within contemporary enhanced-recovery pathways. Our findings support the integration of metronidazole, particularly in its topical form, into a standardised multimodal postoperative protocol.

This review has several limitations. Although only RCTs were included, many had small sample sizes, with almost half enrolling fewer than 50 patients. Considerable heterogeneity was present (*I*^2^ > 80% for several outcomes), likely reflecting differences in surgical technique (open vs. closed haemorrhoidectomy), dosing regimens, and supplementary analgesic use. Risk of bias assessment identified concerns in over half of the included studies, particularly regarding reporting and randomisation methods. Furthermore, adverse effects were poorly documented. Finally, none of the included trials evaluated long-term outcomes such as wound healing, recurrence, or microbiome disruption. Female representation was lacking in at least one study [24].

Despite these limitations, the evidence strongly suggests that metronidazole reduces pain after haemorrhoidectomy, with topical formulations providing early benefit and avoiding systemic exposure.

## Conclusion

In conclusion, this review synthesises the best available evidence demonstrating that metronidazole is effective in reducing pain following excisional haemorrhoidectomy. Both oral and topical formulations are beneficial; however, topical administration appears to provide a superior analgesic effect from post-operative day three and may offer improved tolerability by minimising systemic adverse effects.

## Supplementary Information

Below is the link to the electronic supplementary material.

ESM 1 (DOCX 269 KB)

## Data Availability

The data underlying this article are available from the corresponding author upon reasonable request.

## References

[1] Hong YS, Jung KU, Rampal S, Zhao D, Guallar E, Ryu S et al (2022) Risk factors for hemorrhoidal disease among healthy young and middle-aged Korean adults. Sci Rep 12(1):129 34996957 10.1038/s41598-021-03838-z PMC8741788

[2] Lohsiriwat V (2012) Hemorrhoids: from basic pathophysiology to clinical management. World J Gastroenterol 18(17):2009–2017 22563187 10.3748/wjg.v18.i17.2009 PMC3342598

[3] De Marco S, Tiso D (2021) Lifestyle and risk factors in hemorrhoidal disease. Front Surg 8:729166 34485376 10.3389/fsurg.2021.729166 PMC8416428

[4] Heah S-M (2004) Acute management of haemorrhoids. Acute Surgical Management:461

[5] Lohsiriwat V, Jitmungngan R (2022) Strategies to reduce post-hemorrhoidectomy pain: a systematic review. Medicina (Kaunas). 10.3390/medicina58030418 35334594 10.3390/medicina58030418 PMC8955987

[6] Lyons NJR, Cornille JB, Pathak S, Charters P, Daniels IR, Smart NJ (2017) Systematic review and meta-analysis of the role of metronidazole in post-haemorrhoidectomy pain relief. Colorectal Dis 19(9):803–811 28589634 10.1111/codi.13755

[7] Carapeti EA, Kamm MA, McDonald PJ, Phillips RK (1998) Double-blind randomised controlled trial of effect of metronidazole on pain after day-case haemorrhoidectomy. Lancet 351(9097):169–172 9449871 10.1016/S0140-6736(97)09003-X

[8] Reinhardt T, Lee KM, Niederegger L, Hess CR, Sieber SA (2022) Indolin-2-one nitroimidazole antibiotics exhibit an unexpected dual mode of action. ACS Chem Biol 17(11):3077–3085 36259427 10.1021/acschembio.2c00462 PMC9679994

[9] Solorio-López S, Palomares-Chacón UR, Guerrero-Tarín JE, González-Ojeda A, Cortés-Lares JA, Rendón-Félix J, et al. (2015) Efficacy of metronidazole versus placebo in pain control after hemorrhoidectomy. Results of a controlled clinical trial. Rev Esp Enferm Dig. 107(11):681–5. 10.17235/reed.2015.3926/2015 26541658

[10] Page MJ, McKenzie JE, Bossuyt PM, Boutron I, Hoffmann TC, Mulrow CD et al (2021) The PRISMA 2020 statement: an updated guideline for reporting systematic reviews. Syst Rev 10(1):89 33781348 10.1186/s13643-021-01626-4 PMC8008539

[11] A E, Z F, H H, I I, M K, M O. Rayyan: a systematic reviews web app for exploring and filtering searches for eligible studies for Cochrane Reviews, p 9

[12] Katz J, Melzack R (1999) Measurement of pain. Surg Clin North Am 79(2):231–252 10352653 10.1016/s0039-6109(05)70381-9

[13] Higgins JPT, Altman DG, Gøtzsche PC, Jüni P, Moher D, Oxman AD et al (2011) The Cochrane Collaboration’s tool for assessing risk of bias in randomised trials. BMJ 343:d5928 22008217 10.1136/bmj.d5928 PMC3196245

[14] Higgins JPT TJ, Chandler J, Cumpston M, Li T, Page MJ, Welch VA. Cochrane handbook for systematic reviews of interventions: Cochrane; 2023 [updated Aug 2023. version 6.4:[Available from: www.training.cochrane.org/handbook.

[15] Luo D, Wan X, Liu J, Tong T (2018) Optimally estimating the sample mean from the sample size, median, mid-range, and/or mid-quartile range. Stat Methods Med Res 27(6):1785–1805 27683581 10.1177/0962280216669183

[16] Wan X, Wang W, Liu J, Tong T (2014) Estimating the sample mean and standard deviation from the sample size, median, range and/or interquartile range. BMC Med Res Methodol 14:135 25524443 10.1186/1471-2288-14-135 PMC4383202

[17] Wilkie BD, Chandra R, Chua J, Lam DCS, Paratz ED, An V et al (2021) Efficacy of postoperative oral metronidazole for haemorrhoidectomy pain: a randomized double-blind, placebo-controlled trial. Colorectal Dis 23(1):274–282 32750730 10.1111/codi.15291

[18] Balfour L, Stojkovic SG, Botterill ID, Burke DA, Finan PJ, Sagar PM. (2002) A randomized, double-blind trial of the effect of metronidazole on pain after closed hemorrhoidectomy. Dis Colon Rectum. 45(9):1186–90; discussion 90–1. 10.1007/s10350-004-6390-y 12352234

[19] Nicholson TJ, Armstrong D (2004) Topical metronidazole (10 percent) decreases posthemorrhoidectomy pain and improves healing. Dis Colon Rectum 47(5):711–716 15054681 10.1007/s10350-003-0129-z

[20] Ala S, Saeedi M, Eshghi F, Mirzabeygi P (2008) Topical metronidazole can reduce pain after surgery and pain on defecation in postoperative hemorrhoidectomy. Dis Colon Rectum 51(2):235–238 18176825 10.1007/s10350-007-9174-3

[21] Ng SS-M, Lee JF-Y, Cheung K-Y, Leung K-L, Yiu RY-C, Lau W-Y (2006) Pre-emptive analgesia and metronidazole on post-haemorrhoidectomy pain control. Surg Pract 10(3):102–105

[22] Rabelo FEF, Lacerda-Filho A, Mansur ES, de Oliveira FH, de Queiroz FL, França-Neto PR et al (2021) Benefits of flavonoid and metronidazole use after excisional hemorrhoidectomy: a randomized double-blind clinical trial. Tech Coloproctol 25(8):949–955 34057643 10.1007/s10151-021-02465-0

[23] Xia W, Barazanchi AWH, MacFater WS, MacCormick AD, Svirskis D, Sammour T et al (2022) Topical versus oral metronidazole after excisional hemorrhoidectomy: a double-blind randomized controlled trial. Dis Colon Rectum 65(11):1362–1372 34897211 10.1097/DCR.0000000000002163

[24] Neogi P, Sinha A, Singh M (2018) Is metronidazole a panacea for post-hemorrhoidectomy pain? Int Surg J 5:3598

[25] Abbas ST, Raza A, Muhammad Ch I, Hameed T, Hasham N, Arshad N (2020) Comparison of mean pain score using topical and oral metronidazole in post milligan morgan hemorrhoidectomy patient; a randomized controlled trial. Pak J Med Sci 36(5):867–871 32704254 10.12669/pjms.36.5.1796 PMC7372682

[26] Munawar Islam S, Zafar M, Azhar M, Ali M, Ali H (2023) The efficacy of oral metronidazole in reducing pain post- hemorrhoidectomy: a randomized controlled trial. Medical Forum Monthly

[27] Razzaq S, Khan Z, Mahmood MA, Khan MN, Iqbal W, Zareen N (eds) (2020) Comparison of effectiveness of topical and oral metronidazole for reducing postoperative pain after hemorrhoidectomy. Medical Forum Monthly

[28] Rooh Ali G, Sughra P, Dileep K, Kanwal H, Abdul W, Jahangir Ali S (2023) Comparative study of placebo versus metronidazole as a role of pain relief post hemorrhoidectomy. Annals of PIMS-Shaheed Zulfiqar Ali Bhutto Medical University 19(2):90–93

[29] Al-Mulhim AS, Ali AM, Al-Masuod N, Alwahidi A (2006) Post hemorrhoidectomy pain. A randomized controlled trial. Saudi Med J 27(10):1538–1541 17013479

[30] Abdullah AAK, Panthi R, Devangan M. (2024) Comparative study between oral and topical metronidazole in early recovery from pain after open haemorrhoidectomy. International Journal of Science and Research.

[31] Firdous F, Arsalan S, Siddique S, Shahzad R, Rasheed G, Khan JS (2025) Comparison of 10% topical metronidazole versus oral metronidazole in hemorrhoidectomy in terms of post-operative pain. Rawal Med J 50(3):640

[32] de Paula PR, Speranzini MB, Hamzagic HC, Bassi DG, Chacon-Silva MA, Novo NF et al (1991) Bacteriology of the anal wound after open hemorrhoidectomy. Qualitative and quantitative analysis. Dis Colon Rectum 34(8):664–669 1855423 10.1007/BF02050347

[33] Rizzo A, Paolillo R, Guida L, Annunziata M, Bevilacqua N, Tufano MA (2010) Effect of metronidazole and modulation of cytokine production on human periodontal ligament cells. Int Immunopharmacol 10(7):744–750 20399284 10.1016/j.intimp.2010.04.004

[34] Suárez LJ, Arce RM, Gonçalves C, Furquim CP, Santos NCD, Retamal-Valdes B et al (2024) Metronidazole may display anti-inflammatory features in periodontitis treatment: a scoping review. Mol Oral Microbiol 39(4):240–259 38613247 10.1111/omi.12459

[35] Volkow ND, Blanco C (2021) The changing opioid crisis: development, challenges and opportunities. Mol Psychiatry 26(1):218–233 32020048 10.1038/s41380-020-0661-4 PMC7398847

[36] Wanis KN, Emmerton-Coughlin HM, Coughlin S, Foley N, Vinden C (2017) Systemic metronidazole may not reduce posthemorrhoidectomy pain: a meta-analysis of randomized controlled trials. Dis Colon Rectum. 10.1097/DCR.0000000000000792 28267013 10.1097/DCR.0000000000000792

[37] Bikfalvi A, Faes C, Freys SM, Joshi GP, Van de Velde M, Albrecht E.(2023) PROSPECT guideline for haemorrhoid surgery: a systematic review and procedure-specific postoperative pain management recommendations. European Journal of Anaesthesiology and Intensive Care. 2(3):e0023 10.1097/EA9.0000000000000023 PMC11783633 39917290

[38] Sammour T, Barazanchi AW, Hill AG (2017) Evidence-based management of pain after excisional haemorrhoidectomy surgery: a PROSPECT review update. World J Surg 41(2):603–614 27766395 10.1007/s00268-016-3737-1

[39] Jin JZ, Xia W, Gao R, Vandal AC, Weston M, Israel L et al (2024) A randomized controlled trial of topical analgesia posthemorrhoidectomy (TAPH trial). Dis Colon Rectum 67(9):1158–1168 38871679 10.1097/DCR.0000000000003419

